# Long-Term Cardiovascular Mortality among 80,042 Older Patients with Bladder Cancer

**DOI:** 10.3390/cancers14194572

**Published:** 2022-09-21

**Authors:** Tianwang Guan, Miao Su, Zehao Luo, Weien Peng, Ruoyun Zhou, Zhenxing Lu, Manting Feng, Weirun Li, Yintong Teng, Yanting Jiang, Caiwen Ou, Minsheng Chen

**Affiliations:** 1 Laboratory of Heart Center, Department of Cardiology, Zhujiang Hospital, Southern Medical University, Guangzhou 510280, China; 2 Guangdong Provincial Biomedical Engineering Technology Research Center for Cardiovascular Disease, Guangzhou 510280, China; 3 Sino-Japanese Cooperation Platform for Translational Research in Heart Failure, Guangzhou 510280, China; 4 Department of Neurology, The First Affiliated Hospital, Sun Yat-sen University, Guangzhou 510080, China; 5 Department of Clinical Medicine, Clinical Medical School, Guangzhou Medical University, Guangzhou 510180, China; 6 Dongguan Hospital of Southern Medical University, Southern Medical University, Dongguan 523059, China

**Keywords:** bladder cancer, cardiovascular disease death, cardio-oncology, older patients, SEER

## Abstract

**Simple Summary:**

We conducted a large-scale population-based study with long-term follow-up to obtain a comprehensive assessment of death causes, especially cardiovascular disease death, among 80,042 older bladder cancer patients from a national cancer registry containing 44 years of data. To our knowledge, this was the first study to report the importance of CVD-related death as a competing risk among older patients with bladder cancer. CVD-related death surpassed BC as the leading cause of death 5–10 years after diagnosis among older BC patients, especially for patients with localized-stage and low-grade tumors. Furthermore, older BC patients had a higher risk of CVD-related death than the general population. Although BC management should be the primary focus of older BC patients, our results emphasized the importance of competing risks, the most prominent being CVD. Individual follow-up and management should focus not only on primary cancer but also on CVD-related death to minimize the risk of death in older patients with bladder cancer.

**Abstract:**

Background: To identify the risk of death from cardiovascular disease (CVD) in older patients with bladder cancer (BC). Methods: This population-based study included 80,042 older BC patients (≥65 years) diagnosed between 1975 and 2018, with a mean follow-up of 17.2 years. The proportion of deaths, competing risk models, standardized mortality ratio (SMR), and absolute excess risk (AER) per 10,000 person-years were applied to identify the risk of CVD-related deaths among older BC patients. Results: For older patients with BC, CVD-related death was the chief cause of death, and cumulative CVD-related mortality also exceeded primary BC as the leading cause of death mostly 5–10 years after BC diagnosis, especially in localized-stage and low-grade subgroups. The risk of short- and long-term CVD-related death in older BC patients was higher than in the general older adult population (SMR = 1.30, 95% CI 1.28–1.32; AER = 105.68). The risk of sex-specific CVD-related deaths also increased compared to the general population of older adults, including heart disease, cerebrovascular diseases, hypertension without heart disease, atherosclerosis, aortic aneurysm and dissection, and other diseases of the arteries, arterioles, and capillaries. Conclusions: CVD-related death is an important competing risk among older BC patients and has surpassed primary BC as the chief cause of death, mainly 5–10 years after BC diagnosis. The risk of CVD-related death in older patients with BC was greater than in the general population. The management of older patients with BC should focus not only on the primary cancer but also on CVD-related death.

## 1. Introduction

Bladder cancer (BC) is one of the 10 most frequently diagnosed malignancies worldwide [1]. Given its prevalence and lengthy management, cancer care costs for BC patients are among the highest [2,3]. Individuals aged ≥65 years account for ~81% of BC cases [4]. The medical burden of older BC patients has increased with the aging population due to the growing proportion of older adults. Identifying the chief cause of death in older patients with BC is the key step in effectively improving prognosis.

Cardiovascular disease (CVD) is responsible for the most deaths worldwide [5]. Increasing concerns about CVD-related death among older patients with BC are fueled by the impact of aging but also by shared CVD risk factors and the cardiovascular toxicity of antitumor therapy [6,7]. However, the risk of CVD-related death in older individuals with BC is still unclear.

Previous studies have focused on the primary cancer cause of BC death but have overlooked the risk of CVD-related death in older BC patients [8]. Recent European Association of Urology Guidelines lack recommendations on the risk of CVD-related death in older patients with BC and focus only on cancer-related deaths [9,10]. Although current studies have reported higher proportions of noncancer deaths (including CVD-related deaths) among all BC patients [11,12,13,14,15,16] and nonmetastatic BC patients [17], such studies have not compared the transforming trend of the competing risks of CVD and BC-related death. Furthermore, because these studies lack stratification according to different age subgroups, their results may not be generalizable to older BC patients [11,12,17]. Most studies have simply delineated the composite CVD death outcome or specific CVD death outcomes, such as fatal heart disease [11,13]. These results on the transforming trend of the competing risk of CVD and BC death are controversial [11,13]. Whether CVD death exceeds BC death to become the leading cause of death in older BC patients is still unclear. Therefore, the identification and quantification of the CVD-related death risk among older BC patients are warranted.

Our study characterized the competing risk of CVD-related death among older patients with BC. We identified BC subgroups at high risk of CVD-related death and quantified the risk of short- and long-term CVD mortality for older BC patients compared with the general population. These findings could provide important population-level data to aid the follow-up and management of older BC patients.

## 2. Materials and Methods

### 2.1. Data Collection

Data from the Surveillance, Epidemiology, and End Results (SEER) database were used in our study. The SEER program is an authoritative source of high-quality cancer registries worldwide [18] and relies on systematic, standardized, and regular data collection procedures for quality assurance and the avoidance of surveillance bias [19,20] (Supplementary File S1: Supplementary Methods). Ethics committee approval was waived due to the use of de-identified data [21].

Older patients diagnosed with BC from 1975 to 2018 were included in our study (Supplementary Figure S1). Older BC patients refer to patients aged ≥65 years [4,22]. The inclusion criteria were (1) case selection (site and morphology, primary site-labeled) = “C67.0–9”; (2) histological diagnosis from 1975 to 2018; (3) active follow-up with the definite cause of death; and (4) only one primary cancer. The exclusion criteria were (1) unknown race; (2) age at diagnosis <65 years; and (3) follow-up <2 months. The BC patients less than 65 years is shown in the File S1.

### 2.2. Study Outcomes

The underlying causes of death in the SEER database were documented using the codes of the International Classification of Diseases 10 (ICD-10) of the National Cancer for Health Statistics [23,24]. The primary outcome was death from any cause, with the cause classified as primary neoplasm, CVD, other neoplasms, and other non-neoplasms, among which CVD and other non-neoplasm causes consisted of six and eight specific causes, respectively (Supplementary Table S1). The person-years of follow-up were cumulated from the initial diagnosis of BC until loss-to-follow-up, date of death, or final follow-up date (31 December 2018).

### 2.3. Statistical Analyses

Categorical variables in the baseline characteristics were evaluated using the chi-squared test [25]. To determine the most common cause of death, the proportion of deaths was defined as the number of a specific cause of death divided by the overall number of all deaths in the individuals with BC. To further assess the interaction between BC and CVD-related deaths, we calculated cumulative mortality using competing risk models [26]. The standardized mortality ratio (SMR) was applied to quantify the risk of CVD-related death in BC patients. The SMR was calculated as the ratio of observed deaths to the number of expected deaths [11,24]. Absolute excess risk (AER) was calculated as follows: AER = 10,000 ([number observed–number expected]/[person-years at risk]). CVD-related AER reflected the absolute increase in CVD death risk (i.e., the CVD death burden) in the population [27]. All statistical analyses were completed with R software (version 3.4.4; Vienna, Austria) and SEER*Stat (version 8.3.9, National Cancer Institute, USA). Statistical significance was defined by a *p*-value < 0.05 (File S1).

## 3. Results

### 3.1. Patient Characteristics

Overall, 80,042 older patients with BC diagnosed from 1975 to 2018 were enrolled (Table 1). Of these, 72.0% were male, 91.4% were white, 49.7% had non-muscle-invasive bladder cancer (NMIBC), 69.2% had a localized-stage tumor, 41.8% had low-grade tumors, and 95.2% had received surgery. Compared to BC patients aged 75–84 and >85 years at diagnosis, patients aged 65–74 years were more likely to be male, black, have localized-stage or low-grade tumors, and be diagnosed from 1975 to 1983. The average follow-up time was 17.2 years (SD 1.7 years) for older patients with BC.

### 3.2. Proportion of CVD-Related versus Primary Cancer-Related Deaths

**Figure 1:**
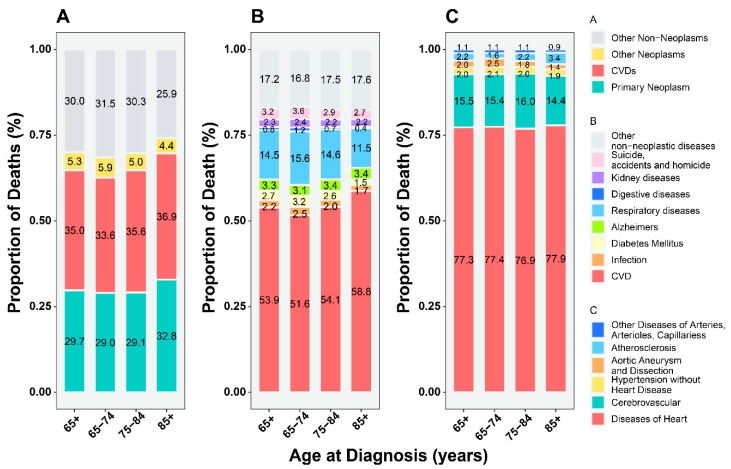
The proportion of deaths among older patients (≥65 years) with bladder cancer. (**A**) All causes of deaths; (**B**) all causes of non-cancer deaths; (**C**) all causes of CVD-related deaths. Abbreviations: CVD, cardiovascular disease.

Older BC patients were likely to die from causes other than BC, chief among which was CVD. When classified by specific CVD, heart disease was the leading cause of death (~70%), while CVD was the main cause of death among noncancer deaths (~50%) (Figure 1). Death from CVD was more common than primary BC among older patients (Figure 1), especially among patients with localized-stage, low-grade tumors and NMIBC (Supplementary Figures S2 and S3A), while the proportion of CVD-related deaths was lower than BC-related deaths in patients <65 years (Supplementary Figure S4).

Stratifying patients by age at diagnosis (Supplementary Figure S5), the proportion of CVD deaths increased with age (65–74, 75–84, and >85 years) and exceeded that of the primary BC-related deaths in men, surgical interventions, white, localized-stage, low-grade, and NMIBC subgroups (Supplementary Figures S3B and S5).

In the follow-up time subgroups (Supplementary Figure S6), the proportion of CVD-related deaths increased markedly with time and exceeded that of primary BC to become the main cause of death 5–10 years after diagnosis in most subgroups, except for the distant-stage subgroup. This transition was observed earlier, 1–5 years after diagnosis, in the localized-stage, low-grade, and NMIBC subgroups (Supplementary Figures S3C and S6).

### 3.3. Cumulative Mortality from the CVD versus Primary Cancer

**Figure 2:**
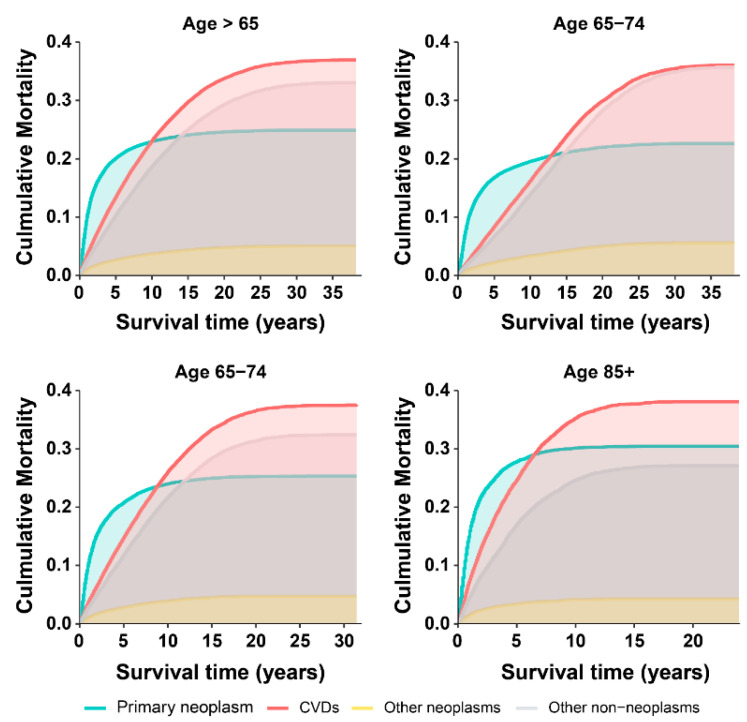
Cumulative mortality among older patients (≥65 years) with bladder cancer.

Cumulative CVD mortality increased steadily with survival time and exceeded primary BC mortality to become the leading cause of death 5–10 years after diagnosis in older BC patients (Figure 2), including the white, other race, localized-stage, surgery, male, low grade, and NMIBC subgroups (Supplementary Figures S3D and S7). No higher incidence of CVD-related deaths was observed other than BC-related deaths in patients <65 years (Supplementary Figure S4). However, this transition was observed earlier (1–5 years after diagnosis) in patients with localized-stage, low-grade tumors and NMIBC (Supplementary Figures S3D and S7), while it was delayed (15 years after diagnosis) in females and the subgroup including other races (Supplementary Figure S7).

To identify patients with a high risk of CVD-related death, further subgroup analyses were conducted, stratifying patients by the age at diagnosis (65–74, 75–84, >85 years) (Supplementary Figure S8). BC patients aged between 65 and 84 years showed higher cumulative mortality from CVD compared with those from primary BC belonging to the following subgroups: males, females, white, other race, localized-stage, low-grade tumor, and surgical intervention. In BC patients aged >85, this transition was observed in the male, white, localized-stage, low-grade tumor, and surgical intervention groups, while the cumulative CVD mortality was close but did not exceed the cumulative BC mortality among females and other races (Supplementary Figure S8). These results were not observed in the other subgroups (Supplementary Figure S9).

### 3.4. CVD Mortality Compared to the General Population

**Figure 3:**
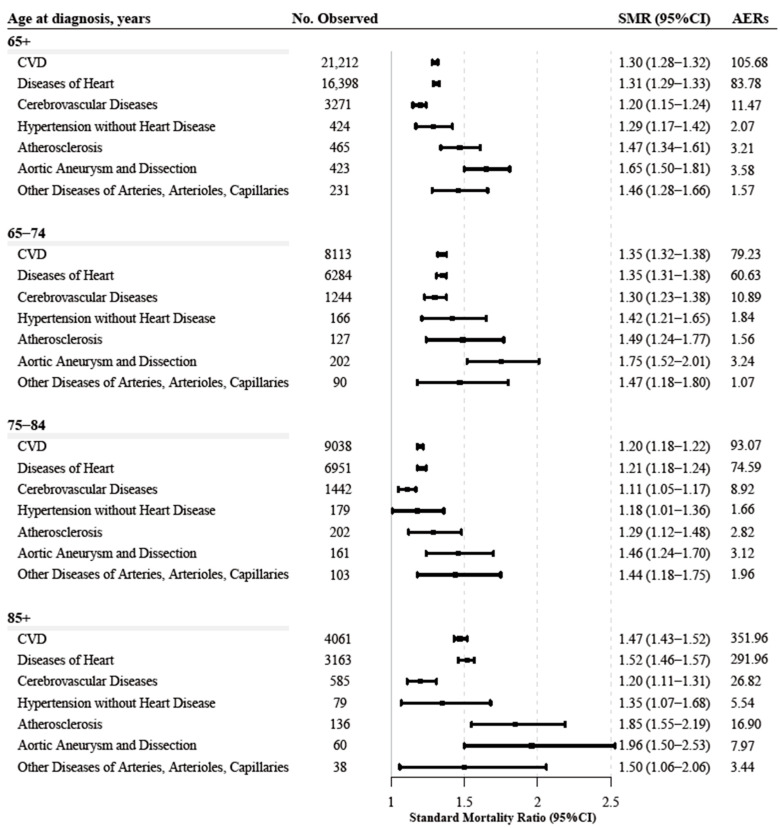
The risk of cardiovascular death among older patients (≥65 years) with bladder cancer.

Compared with the general population, older BC patients had a higher risk of CVD death (SMR = 1.30, 95% CI 1.28–1.32; AER = 105.68), specifically from heart disease, cerebrovascular diseases, atherosclerosis, aortic aneurysm and dissection, hypertension without heart disease, and other diseases of the arteries, arterioles, capillaries. Similar patterns were observed for patients aged >65 years of age at diagnosis (Figure 3). NMIBC and muscle-invasive and metastatic BC (MMIBC) both had higher CVD-related SMR (Supplementary Table S2).

**Figure 4:**
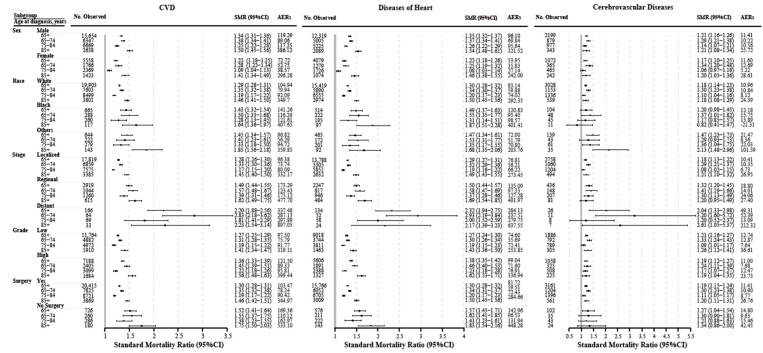
The risk of cardiovascular death among older patients (≥65 years) with bladder cancer according to age at diagnosis. Abbreviations: CVD, cardiovascular disease.

In individualized subgroups, the risks of CVD-related death, heart disease, and cerebrovascular disease were also higher in BC patients aged >65 years compared to the general population (Figure 4). The other four outcomes (atherosclerosis, aortic aneurysm and dissection, hypertension without heart disease, and other diseases of the arteries, arterioles, and capillaries) showed similar trends (Supplementary Figures S10–S13).

In terms of the risk of CVD death according to short-term and long-term follow-up, CVD-related death risk, including the six specific outcomes mentioned above in older-aged BC patients, was greater than in the general population (Table 2; Supplementary Table S3).

## 4. Discussion

This large-scale, population-based, long-term follow-up study performed a comprehensive assessment of the risk of CVD-related death among 80,042 older BC patients using a national cancer registry comprising 44 years of data. To our knowledge, this was the first study to report the importance of CVD-related death as a competing risk among older BC patients. CVD-related death surpassed BC as the leading cause of death 5–10 years after diagnosis among older BC patients, especially for patient subgroups of localized-stage and low-grade tumors. Furthermore, older BC patients had a greater risk of CVD-related death than the general population.

CVD-related deaths surpassed BC-related deaths as the chief cause of death among older BC patients, while the main cause of death in BC patients aged <65 years remained BC. These results were confirmed in terms of the proportion of deaths and by a competing risk model. The proportion of CVD-related deaths was significantly higher than that of BC-related deaths in older patients. Furthermore, the competing risk model analyzing the interaction between CVD-related and BC-related deaths [26] revealed that the cumulative CVD mortality exceeded BC mortality in patients 5–10 years after the BC diagnosis. Our results are supported by the heterogeneity at the cancer site and heterogeneous outcomes in older patients (Figure 1 and Supplementary Figure S4) [28]. In contrast, some studies found that the greatest risk of CVD appeared in the first year after cancer diagnosis [29] and the long-term risk of CVD death might not exceed that of the primary cancer [11]. Previous studies have reported CVD deaths in BC patients; however, these studies did not analyze long-term trends during follow-up and provided a rough analysis of composite outcomes in the general population of BC patients [11,12]. Similar to our results, a population-based study also found that the proportion of noncancer deaths in BC patients gradually exceeded that of cancers with increasing calendar years, although the study did not stratify cases according to the type of CVD-related death or into older-aged subgroups [12]. In partial contrast to our study, a population-based study including all BC patients found a higher incidence of CVD-related deaths than BC-related deaths only in the proportion of deaths analysis but not in the competitive risk model [11], which could be attributed to the rough analysis of the general BC population masking information on important subgroup characteristics specific to older populations.

Considering that analyses including the general population of older BC could mask information about subgroup characteristics, we conducted subgroup analyses among older-aged BC patients. Subgroups with a higher risk of CVD-related deaths than BC-related deaths included the older-aged groups (65–74, 75–84, and >85), white race, other races, male or female, localized-stage, low-grade tumors, surgery, and NMIBC subgroups. Remarkably, in the localized-stage and low-grade subgroup, the higher risk of CVD-related mortality surpassed that of BC-related mortality shortly after BC diagnosis, which was confirmed by the analysis of the proportion of deaths and the competing risk model. Our findings are consistent with a population-based cohort study in which cumulative mortality from noncancer deaths exceeded deaths from cancer in older patients with early-stage Hodgkin lymphoma [27]. Similarly, a breast cancer cohort study also supported our results, finding that CVD competed with breast cancer as the chief cause of death in early-stage and older patients [30]. In contrast, a study using the Cox regression model found that BC patients with localized-stage and low-grade tumors had a lower risk of CVD-related death [14]. This difference might be attributed to differences in the statistical approach used, as indicated by two other studies from the United States [13,31] showing a higher risk of death from heart disease and stroke in cancer patients with localized-stage and low-grade tumors by logistic regression, whereas the results from Cox proportional hazards regression showed the opposite findings. Neither study adequately accounted for competing risks and resulted in biased conclusions, while our competing risk model provided a more reliable framework to analyze the interaction between BC and CVD, as it correctly estimated the marginal probability of an event in the presence of competing events [26]. Further supporting evidence comes from another study using the competing risk model that found that the risk of CVD-related death is higher than cancer-related death in older patients with early-stage breast cancer [32]. Interestingly, aging is a well-known risk factor for CVD and cancer, but current guidelines remain controversial about defining the age cutoff for high-risk CVD death in cancer patients. The European Society of Cardiology (ESC) guidelines proposed that cancer patients >65 years are at high risk of CVD [33], while the consensus of the European Society for Medical Oncology (ESMO) emphasizes that the age cutoff is 75 years [34]. Our findings support this controversy. BC patients ≥65 years were all at higher risk of CVD-related death than BC-related death, but this relationship was not observed in BC patients aged <65 years. In fact, consistent with most previous studies, individuals ≥65 years were defined as the older population and were associated with a high risk of CVD [28]. Moreover, patients ≥65 years formed the largest group of BC patients [4]. Therefore, our results provide a scientific rationale for considering 65 years as the cutoff point for high-risk CVD-related death in older-aged BC patients.

Was the elevated CVD risk in older BC patients influenced by aging or by the direct and indirect effects of BC? The increase in CVD-SMR partially reflects the direct and indirect CVD effects on BC, due to SMR adjusted by age, sex, and ethnicity to the general population at the same time; thus, the results of the SMR largely excluded the effects of aging [13,31]. Our study quantified the risk of CVD death in older BC patients by SMR and found that compared to the general population, older BC patients had a higher risk of CVD-related death, which was also confirmed in clinical subgroups, follow-up time subgroups, and specific CVD types. According to a previous community cohort study, cancer patients had a higher risk of CVD than noncancer controls, but the risk of CVD death was not evaluated [35]. The heterogeneity of the specific causes of CVD deaths was revealed in older patients with BC. Heart disease accounts for the highest proportion of CVD deaths (~70%), followed by cerebrovascular disease (~20%), while the highest CVD-SMR was observed in aortic aneurysm and dissection, followed by atherosclerosis and other diseases of the arteries, arterioles, and capillaries. Similarly, a UK cohort study found that compared to cancer-free controls, BC patients had a 1.18–1.31-fold risk of heart disease and a 1.14-fold risk of stroke [36], offering a possible explanation for our findings. 

Our study offered new insights into the interaction between CVD death and BC death to advance the understanding of cardio-oncology. Although the management of BC should be the primary focus of older BC patients [1,9], our results emphasized the importance of competing risks, chief among which is CVD. The time points associated with a higher risk of CVD-related death than BC-related should guide a multidisciplinary team to focus on CVD-related death risk in older BC patients. Multidisciplinary teams, including a urological surgeon, cardiologist, and cardio-oncologist, could work together to manage and mitigate CVD-related death risk in older BC patients.

The causes of increased CVD-related death risk in older BC patients are diverse, but our analysis suggests that, besides aging, the indirect and direct effects of BC (shared risk factors, anticancer cardiotoxicity, and cancer itself) on CVD likely play an important role. First, aging is associated with a higher risk of CVD-related deaths. Second, shared risk factors for CVD and cancer, such as obesity and smoking, promote the risk of CVD-related death [6]. Third, the fatal cardiotoxicity of chemotherapy and immunotherapy [7] also increases the risk of CVD in older BC patients. Finally, increasing evidence from clinical studies and basic research suggests that cancer may directly damage the cardiovascular system. A new cancer diagnosis is independently related to increased CVD-related death risk [37]. BC can induce arterial and venous thromboembolism, increasing the risk of cardiovascular events, such as cancer-associated stroke [38,39,40]. Cancer itself could damage the heart in treatment-naive individuals with cancer [41]. Cancer-induced neutrophil extracellular traps (NETs) accumulate in the systemic vasculature and heart, resulting in vascular and cardiac dysfunction [42,43]. Nevertheless, the causative contribution of BC itself to cardiovascular disease still needs to be further explored.

The strengths of our study were its large-scale multicenter population and long-term follow-up. Ours is one of the largest studies evaluating the risk of CVD-related death in older BC patients. The size of our study population allowed a comprehensive, stratified analysis by patient characteristics and specific outcomes. Long-term follow-up allowed us to quantify the short- and long-term risk of CVD-related death.

**Study Limitations.** Some limitations to the study should be considered. Despite the larger population size, only a limited number of deaths occurred in some categories (e.g., hypertension without heart disease), as reflected in the imprecise SMR estimates. The SEER database did not provide detailed treatment information, and we could not evaluate the risk of CVD death according to chemotherapeutic agents or immunotherapy. The lack of cardiovascular comorbidities, cardiovascular risk factors, and symptoms such as hematuria [44,45,46] in the SEER database prevents the further exploration of their impact on the risk of CVD death. Nonetheless, the key result in our study is a phenomenal description but not a causal result, while anticancer therapy, cardiovascular risk factors, and comorbidities were majorly associated with the causes of the phenomenon. The SEER database did not have data on residual disease and positive surgical margins’ locations, and we could not further explore their impact on CVD death risk in older BC patients [47,48].

## 5. Conclusions

CVD-related death is an important competing risk among older BC patients and surpasses BC as the leading cause of death 5–10 years after BC diagnosis. The risk of CVD-related death in older patients was higher than in the general older population. Cardio-oncology care, including strategies to prevent, screen, monitor, and treat CVD-related death, is needed for the growing population of older BC patients.

## Figures and Tables

**Table 1:** Baseline characteristics of elderly patients with bladder cancer.

	Age at Diagnosis (*n*/%)				
Characteristic	Total	65–74 Years	75–84 Years	85+ Years	*p* Value
Overall	80,042	35,841	31,676	12,525	
Sex					
Male	57,634 (72.0)	26,926 (75.1)	22,724 (71.7)	7984 (63.7)	<0.001
Female	22,408 (28.0)	8915 (24.9)	8952 (28.3)	4541 (36.3)	
Race					
White	73,151 (91.4)	32,692 (91.2)	29,017 (91.6)	11,442 (91.4)	<0.001
Black	3320 (4.1)	1611 (4.5)	1239 (3.9)	470 (3.8)	
Other *	3571 (4.5)	1538 (4.3)	1420 (4.5)	613 (4.9)	
Stage					
Localized	55,374 (69.2)	25,195 (70.3)	21,808 (68.8)	8371 (66.8)	<0.001
Regional	15,829 (19.8)	6504 (18.1)	6519 (20.6)	2806 (22.4)	
Distant	2702 (3.4)	1300 (3.6)	1072 (3.4)	330 (2.6)	
Unknown	6137 (7.7)	2842 (7.9)	2277 (7.2)	1018 (8.1)	
Grade					
Low	33,495 (41.8)	16,072 (44.8)	12,982 (41.0)	4441 (35.5)	<0.001
High	35,222 (44.0)	14,619 (40.8)	14,375 (45.4)	6228 (49.7)	
Other ^#^	50 (0.1)	14 (0.0)	23 (0.1)	13 (0.1)	
Unknown	11,275 (14.1)	5136 (14.3)	4296 (13.6)	1843 (14.7)	
Subtype					<0.001
NMIBC	39,791 (49.7)	17,670 (49.3)	15,973 (50.4)	6148 (49.1)	
MMIBC	12,249 (15.3)	4793 (13.4)	5127 (16.2)	2329 (18.6)	
Unknown	28,002 (35.0)	13,378 (37.3)	10,576 (33.4)	4048 (32.3)	
Years of diagnosis					
1975–1983	12,457 (15.6)	6027 (16.8)	4787 (15.1)	1643 (13.1)	<0.001
1984–1993	16,459 (20.6)	7902 (22.0)	6379 (20.1)	2178 (17.4)	
1994–2003	17,991 (22.5)	7500 (20.9)	7671 (24.2)	2820 (22.5)	
2004–2018	33,135 (41.4)	14,412 (40.2)	12,839 (40.5)	5884 (47.0)	
Surgery					
Yes	76,202 (95.2)	34,192 (95.4)	30,221 (95.4)	11,789 (94.1)	<0.001
No	3445 (4.3)	1481 (4.1)	1287 (4.1)	677 (5.4)	
Unknown	395 (0.5)	168 (0.5)	168 (0.5)	59 (0.5)	

* Other includes American Indian/Alaska Native and Asian/Pacific Islander. ^#^ Other includes B-cell, pre-B, B-precursor, and B-cell. Abbreviations: NMIBC, non–muscle-invasive bladder cancer; MMIBC, muscle-invasive and metastatic bladder cancer.

**Table 2:** Standardized mortality ratios for cardiovascular death in elderly patients with bladder cancer based on years after diagnosis.

	Years After Diagnosis									
	<1		1–5		5–10		10–15		15+	
Cause of Death	Obs	Smr (95% CI)	Obs	Smr (95% CI)	Obs	Smr (95% CI)	Obs	Smr (95% CI)	Obs	Smr (95% CI)
**65+ years**										
CVD	2686	1.57	7207	1.23	5805	1.28	3244	1.3	2270	1.34
		(1.51–1.63)		(1.20–1.26)		(1.25–1.32)		(1.25–1.34)		(1.28–1.39)
Diseases of the Heart	2124	1.62	5615	1.25	4483	1.29	2472	1.29	1704	1.32
		(1.55–1.69)		(1.21–1.28)		(1.25–1.33)		(1.24–1.35)		(1.25–1.38)
Cerebrovascular Diseases	380	1.34	1075	1.1	906	1.19	523	1.22	387	1.33
		(1.21–1.48)		(1.04–1.17)		(1.11–1.27)		(1.12–1.33)		(1.20–1.46)
Hypertension without Heart Disease	40	1.4	104	1	113	1.25	81	1.41	86	1.82
		(1.00–1.90)		(0.82–1.21)		(1.03–1.50)		(1.12–1.76)		(1.46–2.25)
Atherosclerosis	67	1.83	161	1.34	123	1.42	74	1.68	40	1.46
		(1.42–2.32)		(1.14–1.56)		(1.18–1.69)		(1.32–2.11)		(1.05–1.99)
Aortic Aneurysm and Dissection	46	1.55	172	1.72	123	1.71	53	1.49	29	1.47
		(1.14–2.07)		(1.48–2.00)		(1.42–2.04)		(1.12–1.95)		(0.99–2.12)
Other Diseases of the Arteries, Arterioles, and Capillaries	29	1.78	80	1.44	57	1.3	41	1.64	24	1.38
		(1.19–2.56)		(1.14–1.79)		(0.99–1.69)		(1.17–2.22)		(0.89–2.06)
**65–74 years**										
CVD	632	1.94	2073	1.51	2090	1.4	1628	1.19	1690	1.18
		(1.79–2.10)		(1.45–1.58)		(1.34–1.46)		(1.13–1.25)		(1.12–1.24)
Diseases of the Heart	506	1.92	1637	1.5	1652	1.42	1230	1.17	1259	1.15
		(1.76–2.10)		(1.43–1.57)		(1.35–1.49)		(1.11–1.24)		(1.09–1.21)
Cerebrovascular Diseases	87	2.01	286	1.47	297	1.25	276	1.19	298	1.21
		(1.61–2.48)		(1.30–1.65)		(1.11–1.40)		(1.05–1.34)		(1.08–1.36)
Hypertension without Heart Disease	6	1.4	23	1.19	32	1.3	43	1.48	62	1.56
		(0.52–3.06)		(0.76–1.79)		(0.89–1.84)		(1.07–2.00)		(1.20–2.00)
Atherosclerosis	11	2.97	34	2.01	32	1.57	23	1.06	27	1.19
		(1.48–5.32)		(1.39–2.80)		(1.07–2.22)		(0.67–1.59)		(0.78–1.73)
Aortic Aneurysm and Dissection	10	1.22	69	2.04	64	1.92	35	1.52	24	1.41
		(0.59–2.25)		(1.58–2.58)		(1.48–2.45)		(1.06–2.11)		(0.91–2.10)
Other Diseases of the Arteries, Arterioles, and Capillaries	12	3.6	24	1.71	13	0.85	21	1.5	20	1.36
		(1.86–6.30)		(1.10–2.55)		(0.45–1.45)		(0.93–2.29)		(0.83–2.11)
**75–84 years**										
CVD	1140	1.59	3179	1.08	2733	1.07	1426	1.33	560	2.16
		(1.50–1.69)		(1.04–1.12)		(1.03–1.11)		(1.27–1.41)		(1.98–2.35)
Diseases of the Heart	894	1.63	2462	1.1	2077	1.07	1089	1.34	429	2.18
		(1.53–1.74)		(1.05–1.14)		(1.02–1.12)		(1.26–1.42)		(1.98–2.40)
Cerebrovascular Diseases	169	1.37	502	0.99	462	1.05	223	1.21	86	1.88
		(1.17–1.60)		(0.91–1.08)		(0.96–1.16)		(1.06–1.38)		(1.51–2.33)
Hypertension without Heart Disease	18	1.62	40	0.76	61	1.12	36	1.35	24	3.25
		(0.96–2.57)		(0.55–1.04)		(0.85–1.43)		(0.94–1.87)		(2.08–4.84)
Atherosclerosis	23	1.67	67	1.08	56	1.02	44	2.08	12	2.66
		(1.06–2.51)		(0.83–1.37)		(0.77–1.33)		(1.51–2.80)		(1.38–4.65)
Aortic Aneurysm and Dissection	24	1.71	70	1.44	47	1.42	15	1.27	5	1.88
		(1.10–2.55)		(1.12–1.81)		(1.04–1.88)		(0.71–2.09)		(0.61–4.39)
Other Diseases of the Arteries, Arterioles, and Capillaries	12	1.75	38	1.38	30	1.26	19	1.82	4	1.52
		(0.90–3.05)		(0.98–1.90)		(0.85–1.80)		(1.09–2.84)		(0.41–3.89)
**85+ years**										
CVD	914	1.38	1955	1.27	982	2.01	190	3.04	20	4.39
		(1.29–1.47)		(1.21–1.33)		(1.89–2.14)		(2.63–3.51)		(2.68–6.78)
Diseases of the Heart	724	1.45	1516	1.3	754	2.04	153	3.24	16	4.67
		(1.34–1.56)		(1.24–1.37)		(1.89–2.19)		(2.75–3.80)		(2.67–7.59)
Cerebrovascular Diseases	124	1.05	287	1.06	147	1.72	24	2.17	3	3.63
		(0.87–1.25)		(0.94–1.19)		(1.45–2.02)		(1.39–3.23)		(0.75–10.61)
Hypertension without Heart Disease	16	1.21	41	1.27	20	1.78	2	1.24	0	-
		(0.69–1.96)		(0.91–1.72)		(1.09–2.75)		(0.15–4.49)		-
Atherosclerosis	33	1.72	60	1.45	35	3.01	7	5.45	1	12.9
		(1.19–2.42)		(1.11–1.87)		(2.10–4.19)		(2.19–11.23)		(0.33–71.88)
Aortic Aneurysm and Dissection	12	1.62	33	1.92	12	2.28	3	4.63	0	-
		(0.84–2.83)		(1.32–2.70)		(1.18–3.98)		(0.96–13.53)		
Other Diseases of the Arteries, Arterioles, and Capillaries	5	0.83	18	1.28	14	3.09	1	1.63	0	-
		(0.27–1.93)		(0.76–2.03)		(1.69–5.18)		(0.04–9.07)		-

Abbreviations: CVD, cardiovascular disease; CI, confidence interval; Obs, observed; SMR, standardized mortality ratio.

## Data Availability

The datasets analyzed in this study are publicly available from the SEER database (http://seer.cancer.gov) (accessed on 16 June 2021).

## Supplementary Materials

The following supporting information can be downloaded at: https://www.mdpi.com/article/10.3390/cancers14194572/s1, reference [11,18,19,24,26,27,49]. File S1: Supplementary Methods. Figure S1: Selection of eligible patients and study design; Figure S2: The proportion of death in older patients (≥65 years) with bladder cancer in different subgroups; Figure S3: The risk of CVD-related deaths among older patients with different subtypes of bladder cancer; Figure S4: The proportion of deaths and cumulative mortality in bladder cancer patients under 65 years; Figure S5: The proportion of deaths in elderly patients with bladder cancer based on age at diagnosis; Figure S6: The proportion of deaths among older patients (≥65 years) with bladder cancer based on the years after diagnosis; Figure S7: Cumulative mortality among older patients (≥65 years) with bladder cancer in different subgroups; Figure S8: The cumulative mortality in older patients (≥65 years) with bladder cancer based on age at diagnosis (high competing risk subgroups); Figure S9: The cumulative mortality in older patients (≥65 years) with bladder cancer based on age at diagnosis (low competing risk subgroups); Figure S10: The death risk of hypertension without heart disease in older patients (≥65 years) with bladder cancer based on age at diagnosis; Figure S11: The death risk of atherosclerosis in older patients (≥65 years) with bladder cancer based on age at diagnosis; Figure S12: The death risk of aortic aneurysm and dissection in older patients (≥65 years) with bladder cancer based on age at diagnosis; Figure S13: The death risk of other diseases of the arteries, arterioles, and capillaries in older patients (≥65 years) with bladder cancer based on age at diagnosis; Table S1: The classifications about non-neoplasm causes of death; Table S2: Standardized mortality ratios for cardiovascular death in older patients with different subtypes of bladder cancer; Table S3: Absolute excess risks for cardiovascular death in older patients (≥65 years) with bladder cancer.

## Abbreviations

AER	Absolute excess risk
BC	Bladder cancer
CVD	Cardiovascular disease
CI	Confidence interval
ESC	The European Society of Cardiology
ESMO	The European Society for Medical Oncology
SMR	Standardized mortality ratio
SEER	Surveillance, Epidemiology, and End Result database
