# Supplementary material for: Long-Term Cardiovascular Mortality among 80,042 Older Patients with Bladder Cancer
Source: Cancers (Basel). 2022 Sep 21;14(19):4572. doi: 10.3390/cancers14194572 (PMC9559628; doi:10.3390/cancers14194572)
Supplement: Supplementary file 1 [file cancers-14-04572-s001.zip › cancers-1903911-supplementary.pdf]

Supplementary File S1: Supplementary Methods

List of Supplementary Tables

Supplementary Table S1. The classifications about non-neoplasms causes of death

Supplementary Table S2. Standardized mortality ratios for cardiovascular death in older patients with different subtypes of bladder cancer

Abbreviations: CI, confidence interval; CVD, Cardiovascular Disease; Obs, observed; SMR, standardized mortality ratio. NMIBC, non–muscle-invasive bladder cancer; MMIBC, muscle-invasive and metastatic bladder cancer.

Supplementary Table S3. Absolute excess risks for cardiovascular death in older patients (≥65 years) with bladder cancer

List of Supplementary Figures

Supplementary Figure S1. Selection of eligible patients and study design

Supplementary Figure S2. The proportion of death in older patients (≥65 years) with bladder cancer in different subgroups

Abbreviations: CVD, cardiovascular disease.

Supplementary Figure S3. The risk of CVD-related deaths among older patients with different subtypes of bladder cancer

Abbreviations: NMIBC, non–muscle-invasive bladder cancer; MMIBC, muscle-invasive and metastatic bladder cancer.

Supplementary Figure S4. The proportion of death and cumulative mortality in bladder cancer patients under 65 years

Supplementary Figure S5. The proportion of death in elderly patients with bladder cancer based on age at diagnosis

Abbreviations: CVD, cardiovascular disease.

Supplementary Figure S6. The proportion of deaths among older patients (≥65 years) with bladder cancer based on the years after diagnosis

Abbreviations: CVD, cardiovascular death.

Supplementary Figure S7. Cumulative mortality among older patients (≥65 years) with

bladder cancer in different subgroups

Abbreviations: CVD, cardiovascular disease.

Supplementary Figure S8. The cumulative mortality in older patients (≥65 years) with bladder cancer based on age at diagnosis (High competing risk subgroups)

Supplementary Figure S9. The cumulative mortality in older patients (≥65 years) with bladder cancer based on age at diagnosis (Low competing risk subgroups)

Supplementary Figure S10. The death risk of hypertension without heart disease in older patients (≥65 years) with bladder cancer based on age at diagnosis

Supplementary Figure S11. The death risk of atherosclerosis in older patients (≥65 years) with bladder cancer based on age at diagnosis

Supplementary Figure S12. The death risk of aortic aneurysm and dissection in older patients (≥65 years) with bladder cancer based on age at diagnosis

Supplementary Figure S13 The death risk of other diseases of arteries, arterioles, capillaries in older patients (≥65 years) with bladder cancer based on age at diagnosis

Supplementary File S1. Supplementary Methods

Surveillance, Epidemiology, and End Results (SEER) Program overview

The Surveillance, Epidemiology, and End Results (SEER) Program of the National Cancer Institute (NCI) is an authoritative source of information on cancer incidence and survival in the United States. The SEER Program is the comprehensive source of population-based information in the United States that includes stage of cancer at the time of diagnosis and patient survival data which began in 1973 and is continually evolving. SEER currently collects and publishes cancer incidence and survival data from population-based cancer registries covering approximately 40 percent of the U.S. population to monitor cancer incidence and survival in the population and advance cancer surveillance research. The SEER Program registries routinely collect data on patient which contains information about patient demographics and cancer characteristics, such as sex, age at diagnosis, year of diagnosis, race, marital status, tumor grade and stage, treatment, and patient survival time. The mortality data reported by SEER are provided by the National Center for Health Statistics. The population data used in calculating cancer rates is obtained periodically from the Census Bureau[1].

The SEER-9 was used in this study. The SEER-9 program was supported by the National Cancer Institute (NCI), spanning the years 1975 to 2009, which collects data from 9 regional cancer registries throughout the USA including Connecticut, Hawaii, Iowa, New Mexico, Utah, San Francisco/Oakland, Detroit (Metropolitan), Atlanta (Metropolitan) and Seattle/Puget Sound. It records of 5,530,535 tumors reported cases. Geographic areas were included based on two objectives: (1) to maintain high quality data which represent minority subgroups, and (2) to represent significant epidemiological information of minority subgroups. SEER-9 adheres to two basic principles when conducting audits: auditing high quantity and high risk data[2].

Study population of bladder cancer patients less than 65 years

The bladder cancer (BC) patients less than 65 years were also included. The inclusion criteria were as follows: (1) case selection (site and morphology, primary site-labeled) =

“C67.0-9”; (2) histological diagnosis during 1975-2018; (3) active follow-up and with definite death cause; (4) only one primary cancer; (5) age at diagnosis less than 65 years. The exclusion criteria were as follows: (1) unknown race; (2) follow-up less than 2 months. A total of 40 067 BC patients less than 65 years were included.

Statistical Analyses

The standardized mortality ratios (SMRs), adjusted by age, sex and ethnicity to the general population over the same time, were defined as the ratio of the observed deaths to the expected. The observed deaths represent the observed number of deaths attributed to a specific cause within a specific time frame, while the expected deaths represent the number of deaths which are expected to attribute to the same cause in a demographically similar population within the same time frame. The expected number of deaths was calculated according to the following formula: expected deaths = person-years × mortality rate of specific cause among general population. The SMR, corresponding exact 95% confidence intervals (CIs) and AER were calculated by SEER*Stat [3-6].

Cumulative mortality was calculated by Fine and Gray’s competing risks model, among which the basic descriptive statistic was the cumulative incidence function which assessed the absolute risk of endpoint events over time. This method is better than Kaplan-Meier method and Cox regression analyses when assessing the competing risks data. Kaplan-Meier method and Cox regression analyses will over-estimates the true absolute risk, while competing risk methods provide the appropriate framework to analyze the interplay among different endpoint events, especially BC and cardiovascular disease death in this study and avoid the biased upwards. Competing risk methods were built and evaluated using R packages ‘cmprsk’ (R software version 3.4.4)[7].

References

1. Park HS, Lloyd S, Decker RH*, et al.* Overview of the Surveillance, Epidemiology, and End Results database: evolution, data variables, and quality assurance. Curr Probl Cancer 2012;36(4):183-90.
2. *National Cancer Institute. About the SEER program*.
3. Sung H, Hyun N, Leach CR*, et al.* Association of First Primary Cancer With Risk of Subsequent Primary Cancer Among Survivors of Adult-Onset Cancers in the United States. Jama 2020;324(24):2521-2535.
4. Sturgeon KM, Deng L, Bluethmann SM*, et al.* A population-based study of cardiovascular disease mortality risk in US cancer patients. Eur Heart J 2019;40(48):3889-3897.
5. Dores GM, Curtis RE, Dalal NH*, et al.* Cause-Specific Mortality Following Initial Chemotherapy in a Population-Based Cohort of Patients With Classical Hodgkin Lymphoma, 2000-2016. J Clin Oncol 2020;38(35):4149-4162.
6. *Surveillance Research Program, National Cancer Institute: SEER*Stat software, version 8.3.9.2*.
7. Austin PC, Lee DS, Fine JP. Introduction to the Analysis of Survival Data in the Presence of Competing Risks. Circulation 2016;133(6):601-9.

Supplementary Table S1. The classifications about non-neoplasms causes of death

**Non-neoplasms causes of death**	**Specific classifications**					
**Cardiocerebrovascular diseases**	Aortic Aneurysm and Dissection	Atherosclerosis	Cerebrovascular Diseases	Diseases of Heart	Hypertension without Heart Disease	Other Diseases of Arteries, Arterioles, Capillaries
**Infection**	Other Infectious and Parasitic Diseases including HIV	Septicemia	Tuberculosis	Syphilis		
**Diabetes Mellitus**	Diabetes Mellitus					
**Alzheimer's disease**	Alzheimer's (ICD-9 and 10 only)					
**Respiratory diseases**	Chronic Obstructive Pulmonary Disease and Allied Cond	Pneumonia and Influenza				
**Digestive diseases**	Chronic Liver Disease and Cirrhosis	Stomach and Duodenal Ulcers				
**Kidney diseases**	Nephritis, Nephrotic Syndrome and Nephrosis					
**Suicide, accidents and homicide**	Accidents and Adverse Effects	Homicide and Legal Intervention	Suicide and Self-Inflicted Injury			
**Other non-neoplastic diseases**	Certain Conditions Originating in Perinatal Period	Complications of Pregnancy, Childbirth, Puerperium	Congenital Anomalies	Other Cause of Death	Symptoms, Signs and Ill-Defined Conditions	

Supplementary Table S2. Standardized mortality ratios for cardiovascular death in older patients with different subtypes of bladder cancer

Cause of death	NMIBC			MMIBC		
	**Obs**	**Smr (95% CI)**	**AER**	**Obs**	**Smr (95% CI)**	**AER**
CVD	10,365	1.24 (1.21-1.26)	72.22	1,883	1.57 (1.50-1.65)	174.57
Diseases of Heart	8,074	1.25 (1.23-1.28)	59.57	1,448	1.58 (1.50-1.66)	135.24
Cerebrovascular Diseases	1,583	1.13 (1.08-1.19)	6.8	281	1.39 (1.23-1.56)	20.09
Hypertension without Heart Disease	258	1.18 (1.04-1.34)	1.46	59	2.00 (1.52-2.58)	7.5
Atherosclerosis	146	1.26 (1.06-1.48)	1.08	33	1.85 (1.28-2.60)	3.87
Aortic Aneurysm and Dissection	187	1.50 (1.29-1.73)	2.27	39	2.12 (1.51-2.90)	5.24
Other Diseases of Arteries, Arterioles, Capillaries	117	1.32 (1.09-1.58)	1.03	23	1.82 (1.15-2.73)	2.64

Abbreviations: CI, confidence interval; CVD, Cardiovascular Disease; Obs, observed; SMR, standardized mortality ratio. NMIBC, non–muscle-invasive bladder cancer; MMIBC, muscle-invasive and metastatic bladder cancer.

Supplementary Table S3. Absolute excess risks for cardiovascular death in older patients (≥65 years) with bladder cancer

	Absolute excess risks				
	Years After Diagnosis				
**Cause of death**	**< 1**	**1-5**	**5-10**	**10-15**	**15+**
**65+ years**					
CVD	163.55	70.34	101.12	129.43	190.06
Diseases of Heart	135.71	57.89	79.84	97.64	135.96
Cerebrovascular Diseases	16	5.31	11.52	16.64	31.66
Hypertension without Heart Disease	1.9	0.01	1.79	4.13	12.91
Atherosclerosis	5.08	2.11	2.87	5.2	4.22
Aortic Aneurysm and Dissection	2.74	3.76	4.05	3.04	3.1
Other Diseases of Arteries, Arterioles, Capillaries	2.13	1.27	1.05	2.77	2.22
**65-74 years**					
CVD	110.61	71.91	79.43	63.03	98.21
Diseases of Heart	87.79	55.82	65.1	44.05	62.96
Cerebrovascular Diseases	15.77	9.32	8.01	10.61	20.26
Hypertension without Heart Disease	0.62	0.38	0.99	3.43	8.59
Atherosclerosis	2.64	1.75	1.55	0.32	1.64
Aortic Aneurysm and Dissection	0.66	3.6	4.09	2.92	2.71
Other Diseases of Arteries, Arterioles, Capillaries	3.13	1.02	-0.31	1.71	2.05
**75-84 years**					
CVD	179.89	31.88	42.19	230	751.81
Diseases of Heart	147.05	29.32	30.62	176.59	581.33
Cerebrovascular Diseases	19.55	-0.69	5.39	25.18	100.91
Hypertension without Heart Disease	2.94	-1.67	1.43	5.98	41.56
Atherosclerosis	3.93	0.65	0.26	14.71	18.75
Aortic Aneurysm and Dissection	4.24	2.86	3.1	2.04	5.85
Other Diseases of Arteries, Arterioles, Capillaries	2.18	1.41	1.38	5.5	3.42
**85+ years**					
CVD	288.7	202.12	724.76	1,385.49	2,125.54
Diseases of Heart	258.4	171.1	563.59	1,148.82	1,731.08
Cerebrovascular Diseases	7.03	8.02	90.22	140.63	299.17
Hypertension without Heart Disease	3.17	4.26	12.86	4.24	-19.35
Atherosclerosis	16.02	9.07	34.32	62.08	126.97
Aortic Aneurysm and Dissection	5.31	7.72	9.88	25.55	-5.93
Other Diseases of Arteries, Arterioles, Capillaries	-1.22	1.94	13.9	4.19	-6.41

Abbreviations: CVD, Cardiovascular Disease.

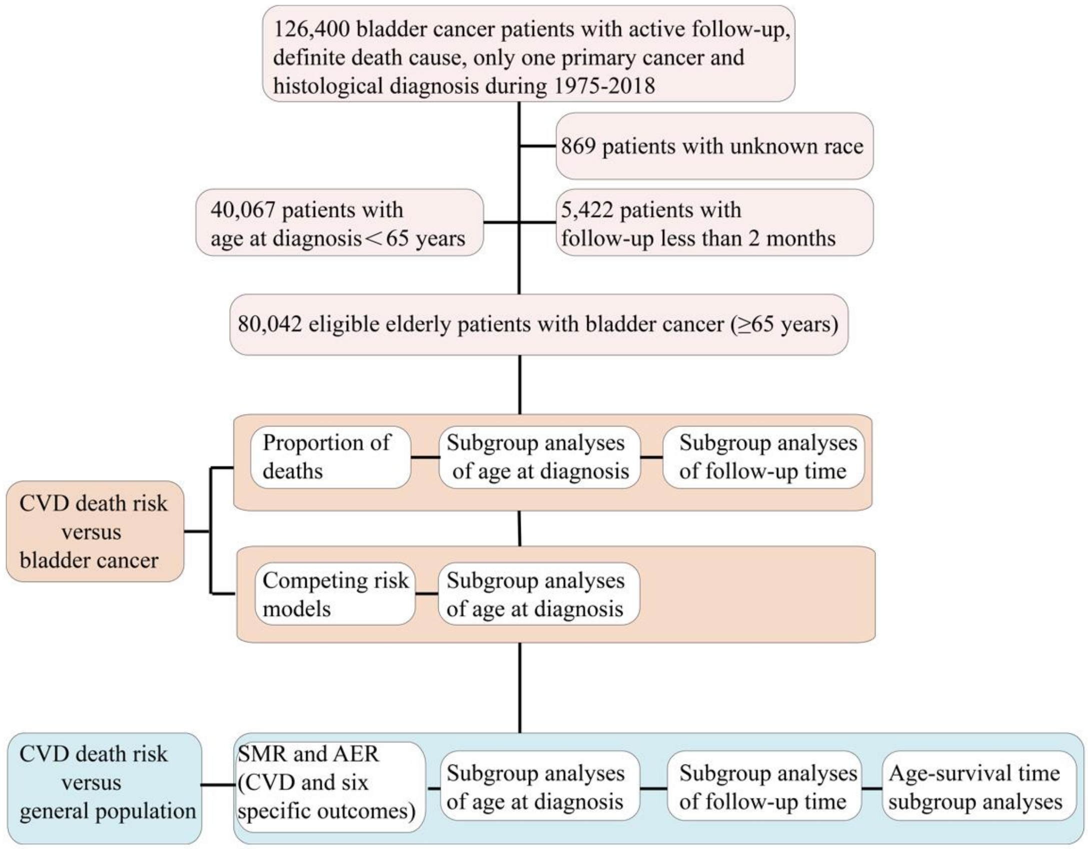

Supplementary Figure S1. Selection of eligible patients and study design

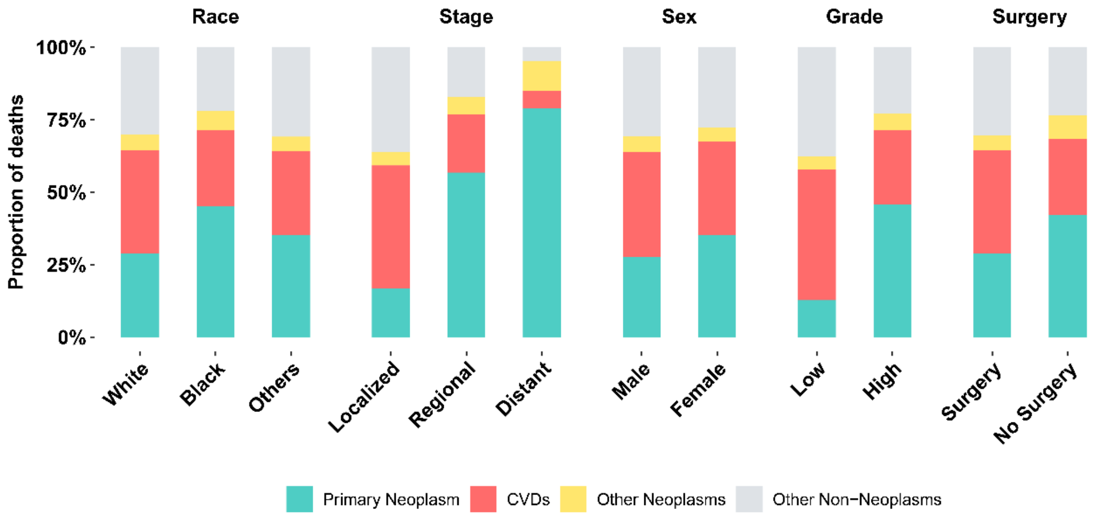

Supplementary Figure S2. The proportion of death in older patients (≥65 years) with bladder cancer in different subgroups

Abbreviations: CVD, cardiovascular disease.

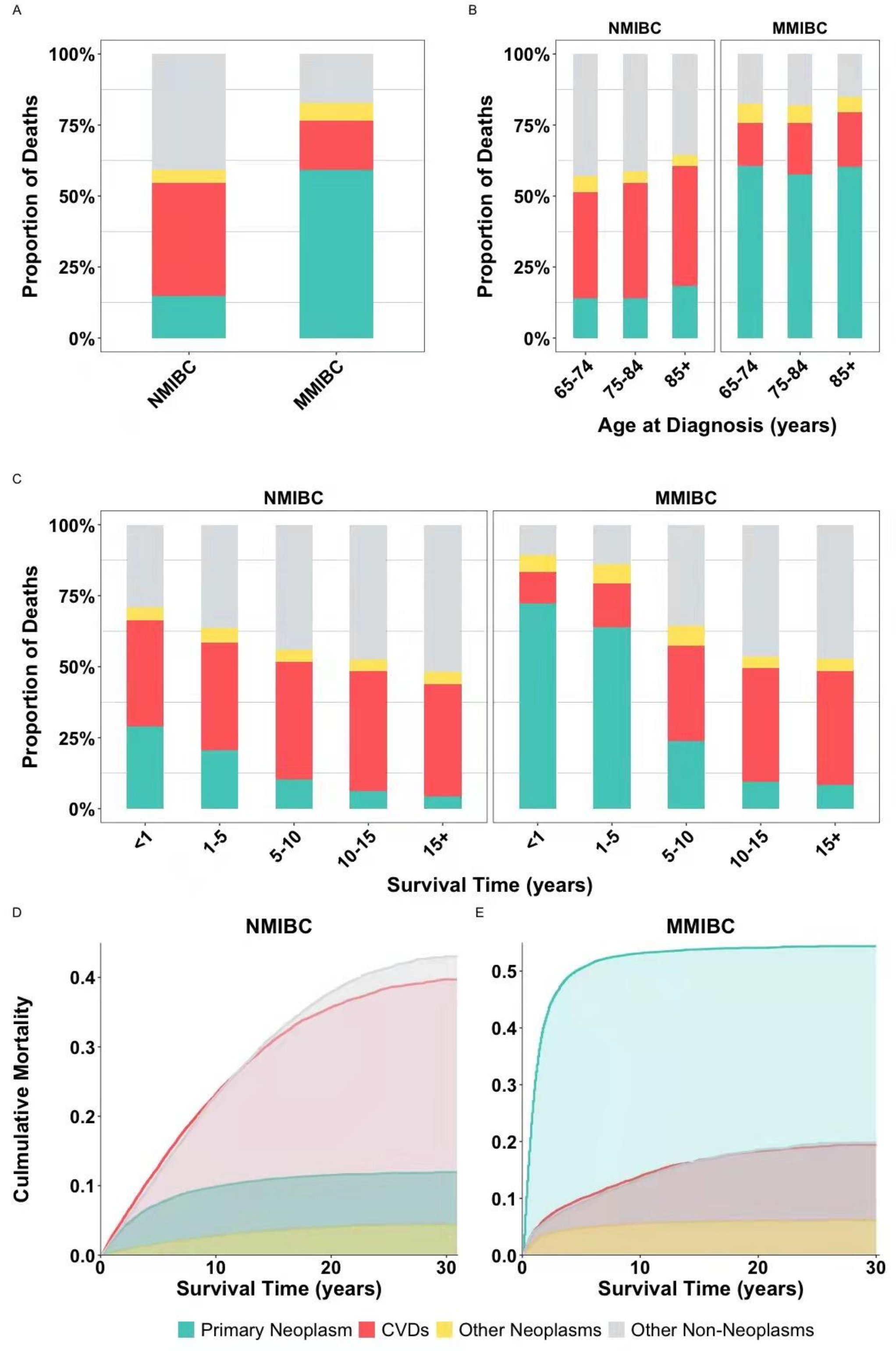

Supplementary Figure S3. The risk of CVD-related deaths among older patients with different subtypes of bladder cancer

Abbreviations: NMIBC, non–muscle-invasive bladder cancer; MMIBC, muscle-invasive and metastatic bladder cancer.

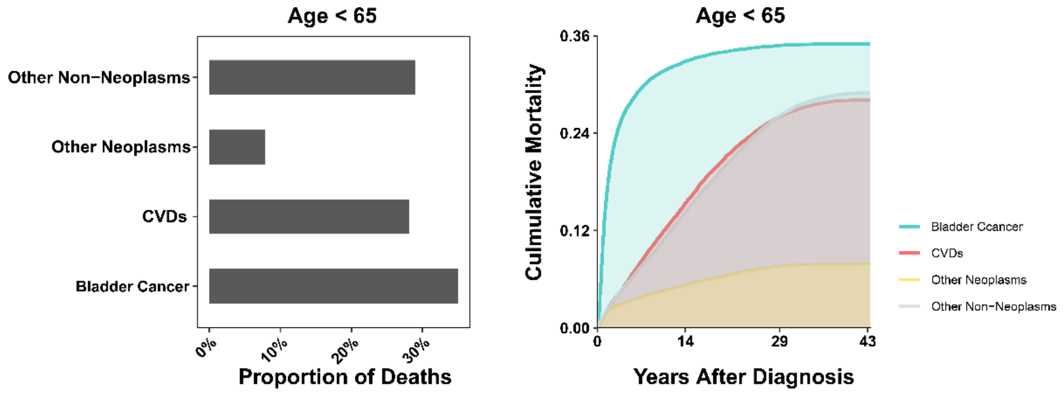

Supplementary Figure S4. The proportion of death and cumulative mortality in bladder cancer patients under 65 years

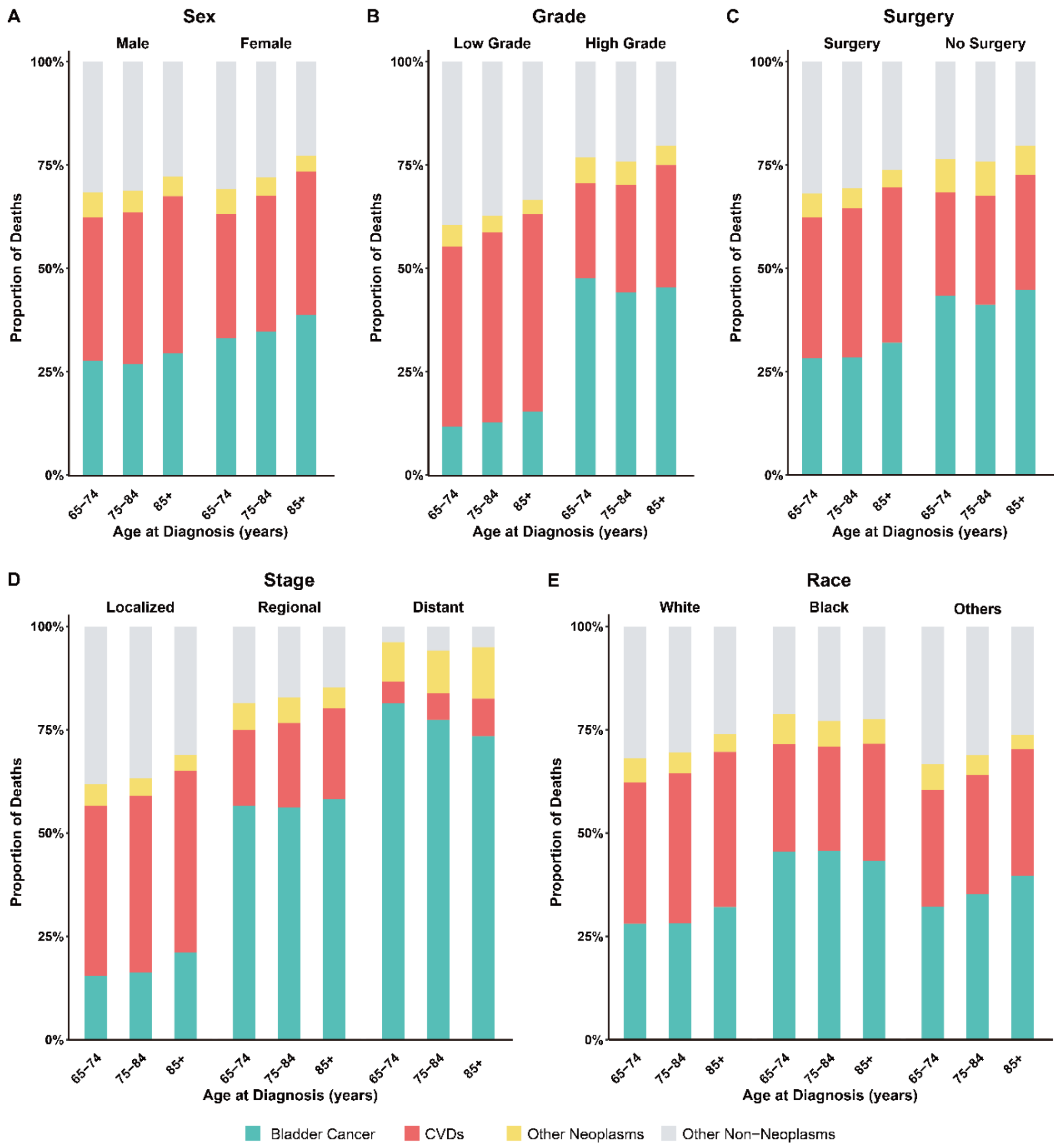

Supplementary Figure S5. The proportion of death in elderly patients with bladder cancer based on age at diagnosis

Abbreviations: CVD, cardiovascular disease.

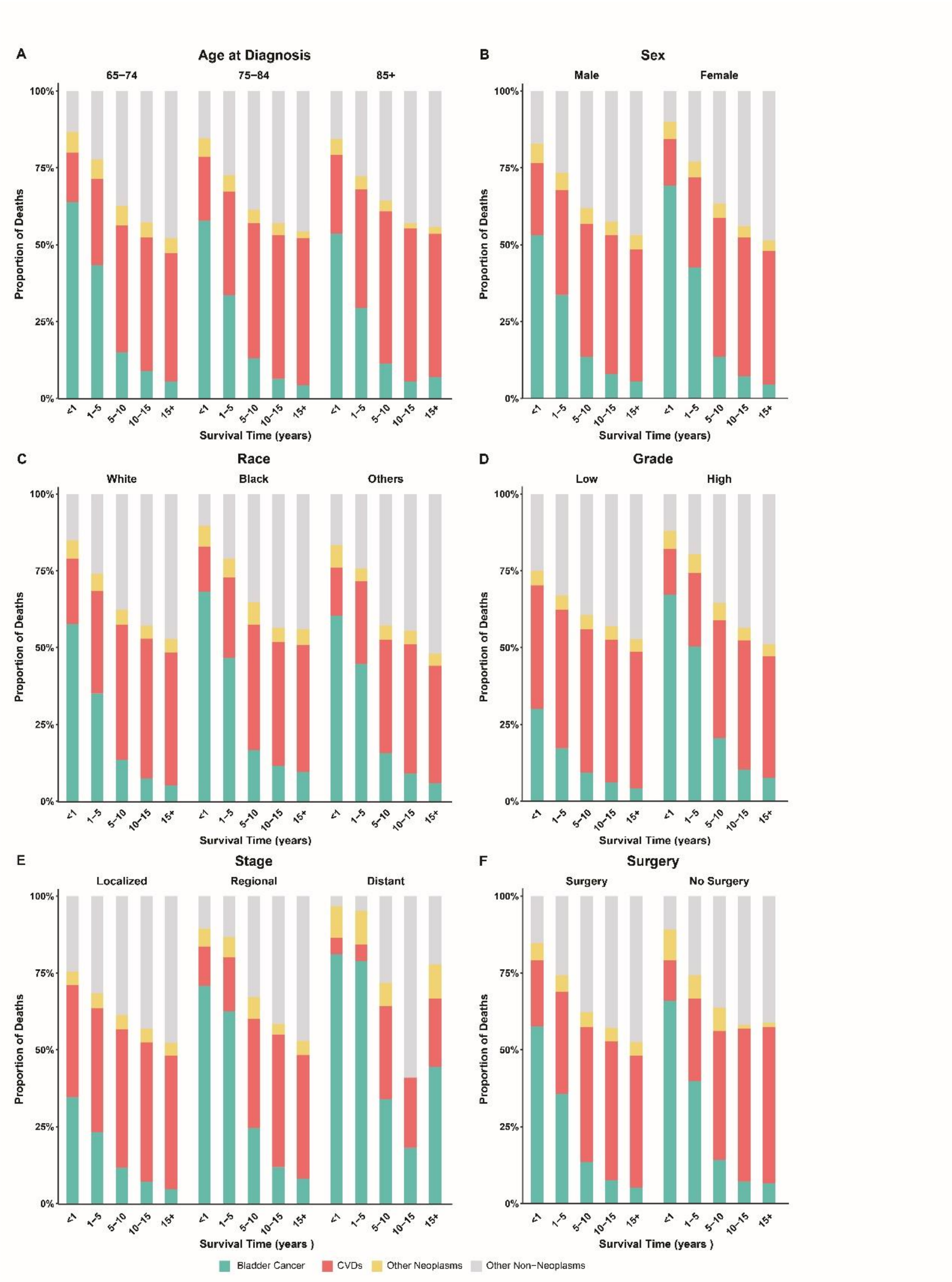

Supplementary Figure S6. The proportion of deaths among older patients (≥65 years) with bladder cancer based on the years after diagnosis

Abbreviations: CVD, cardiovascular death.

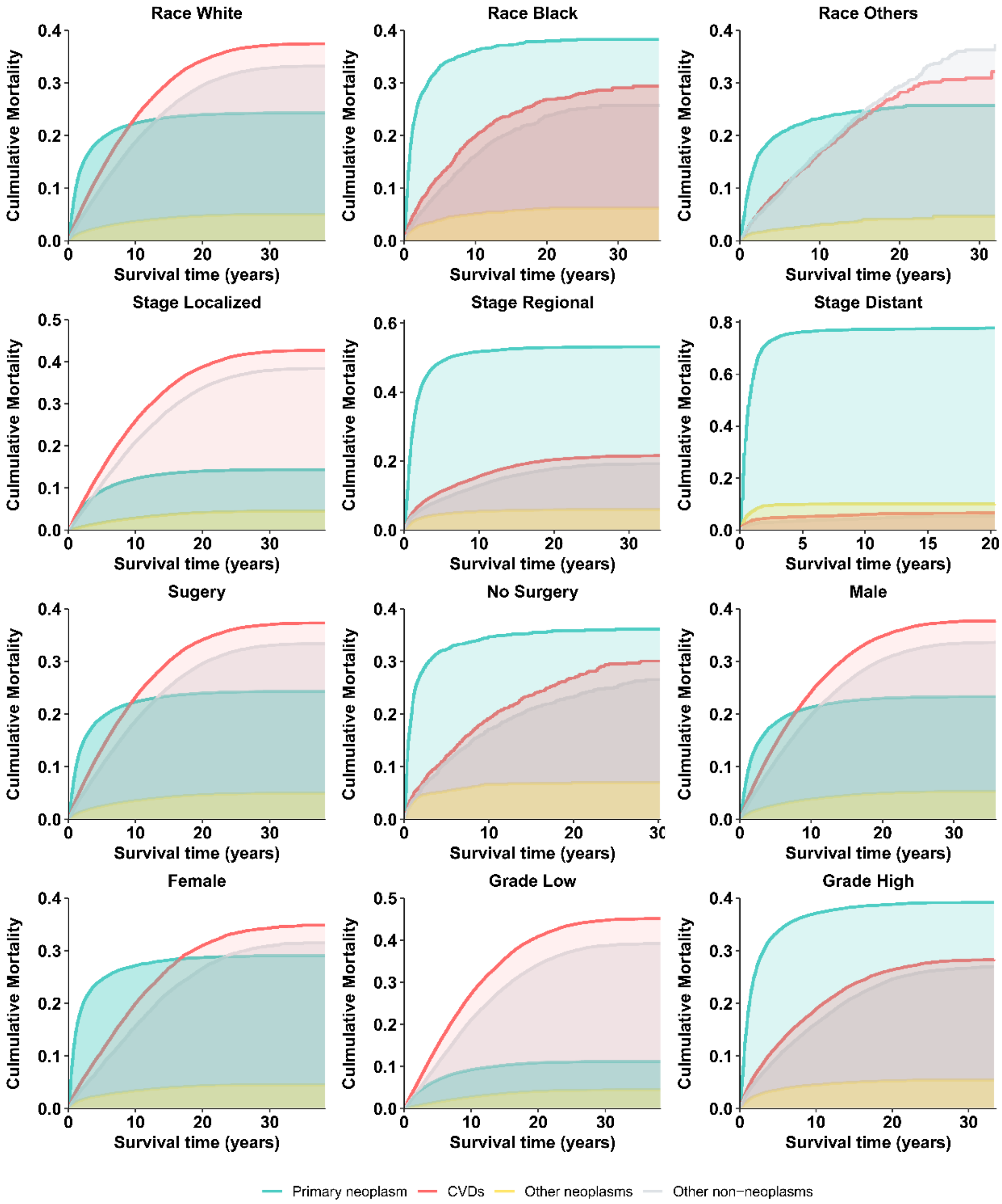

Supplementary Figure S7. Cumulative mortality among older patients (≥65 years) with bladder cancer in different subgroups

Abbreviations: CVD, cardiovascular disease.

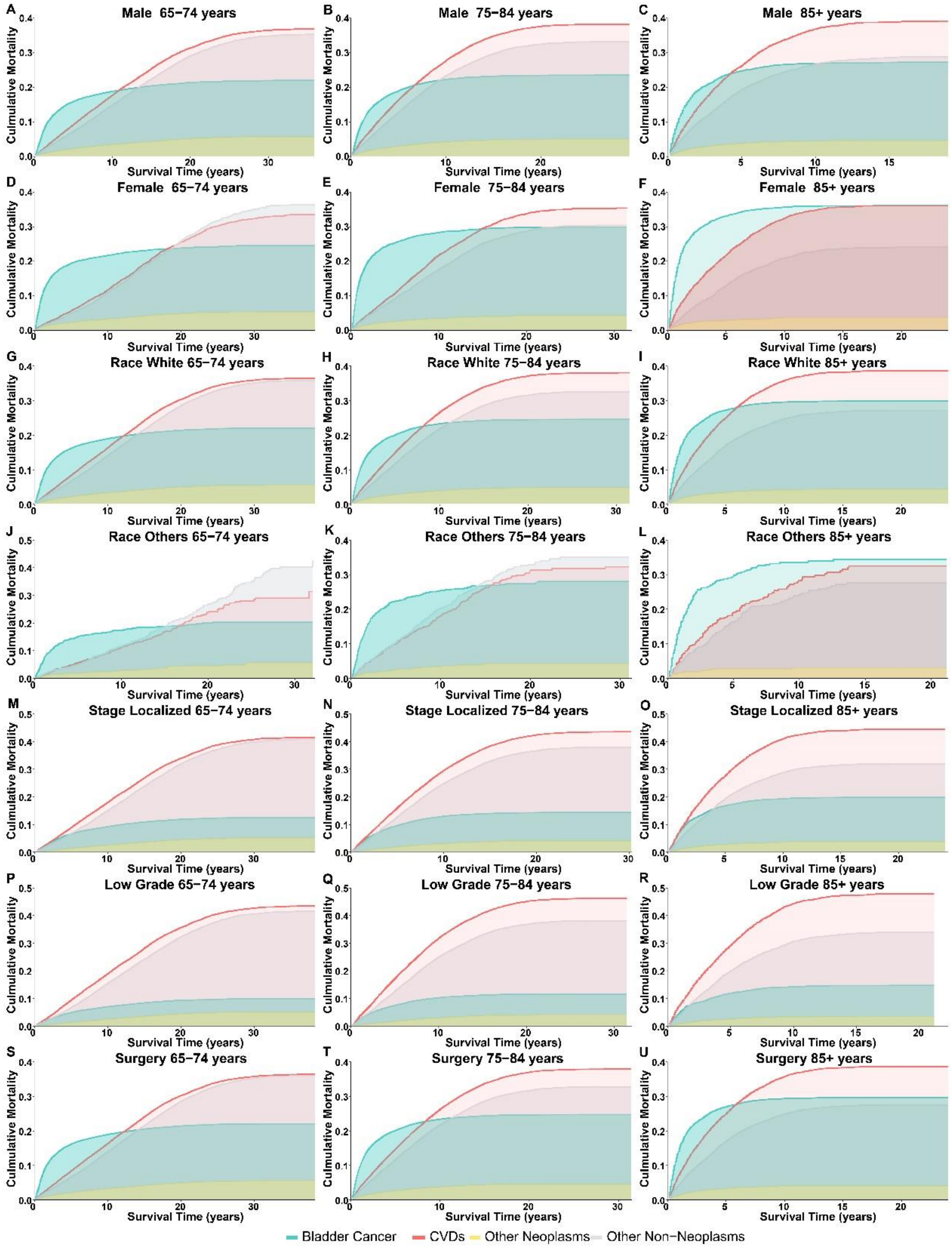

Supplementary Figure S8. The cumulative mortality in older patients (≥65 years) with bladder cancer based on age at diagnosis (High competing risk subgroups)

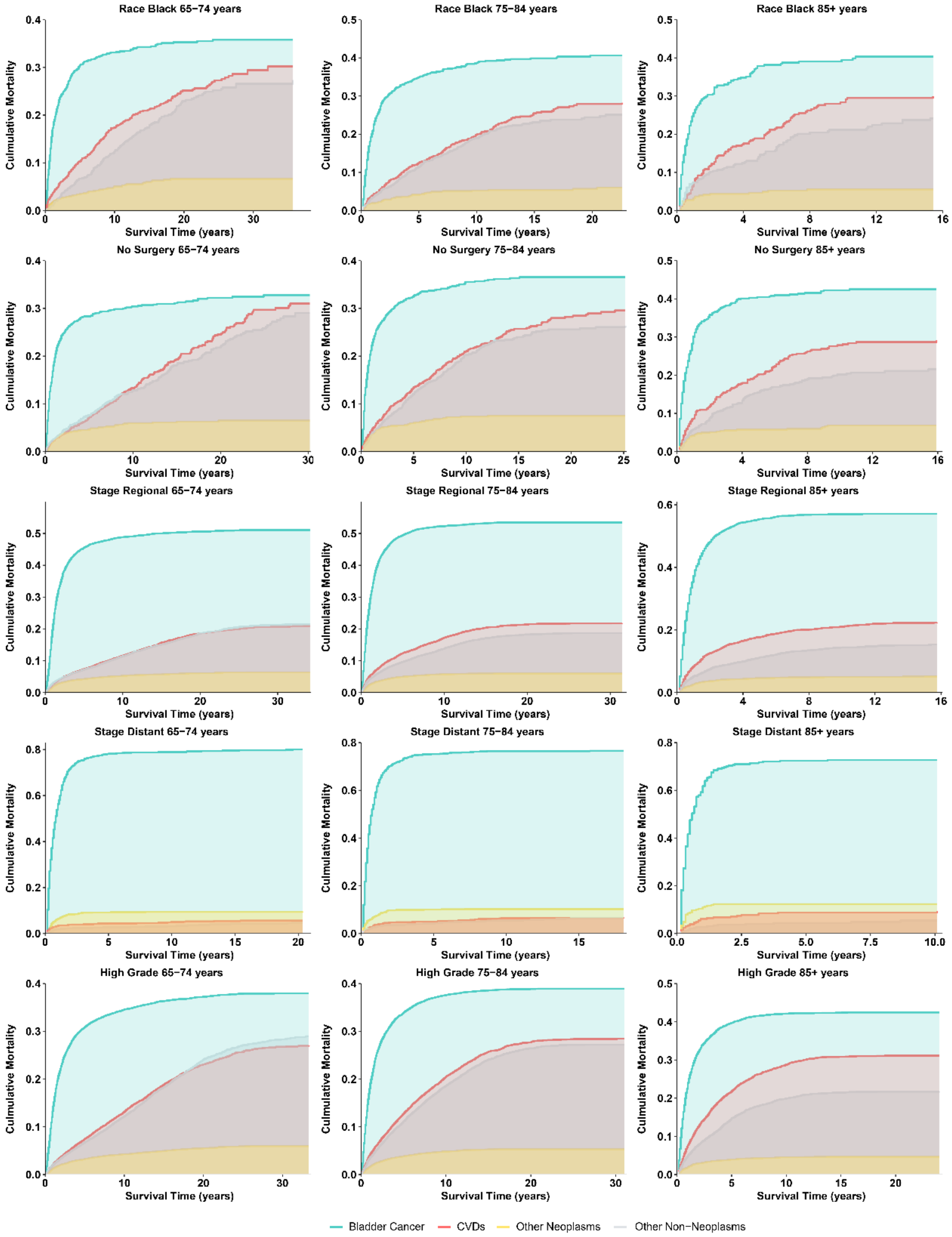

Supplementary Figure S9. The cumulative mortality in older patients (≥65 years) with bladder cancer based on age at diagnosis (Low competing risk subgroups)

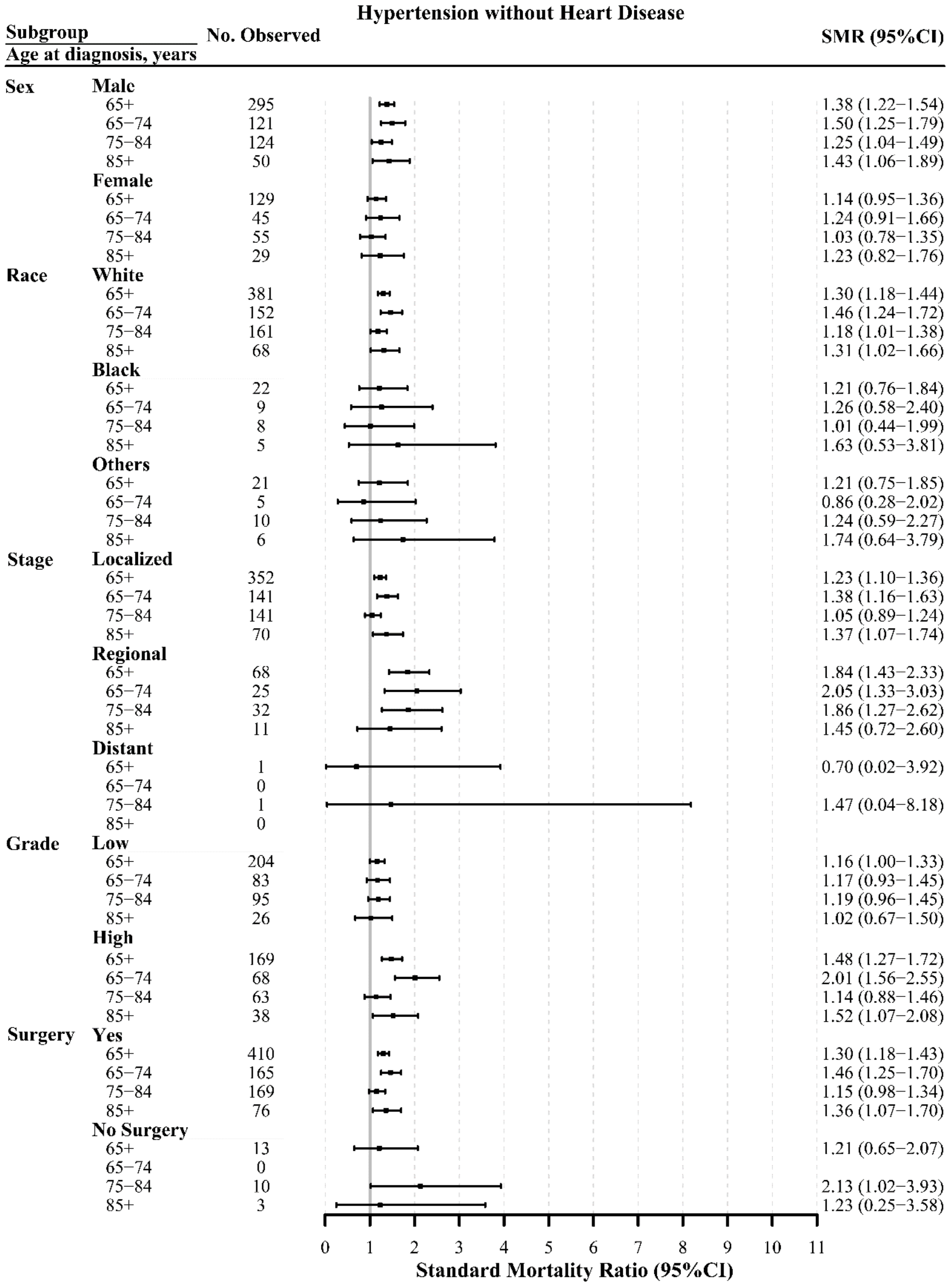

Supplementary Figure S10. The death risk of hypertension without heart disease in older patients (≥65 years) with bladder cancer based on age at diagnosis

Subgroup	Age at diagnosis, years	No. Observed	SMR (95%CI)
**Sex**	**Male**		
	65+	327	1.56 (1.40–1.74)
	65–74	98	1.58 (1.28–1.92)
	75–84	148	1.43 (1.21–1.68)
	85+	81	1.88 (1.49–2.33)
	**Female**		
	65+	138	1.30 (1.09–1.53)
	65–74	29	1.24 (0.83–1.78)
	75–84	54	1.03 (0.77–1.34)
	85+	55	1.81 (1.36–2.36)
**Race**	**White**		
	65+	456	1.50 (1.37–1.65)
	65–74	125	1.53 (1.27–1.82)
	75–84	199	1.32 (1.15–1.52)
	85+	132	1.86 (1.55–2.20)
	**Black**		
	65+	6	0.85 (0.31–1.86)
	65–74	1	0.44 (0.01–2.44)
	75–84	3	0.89 (0.18–2.60)
	85+	2	1.47 (0.18–5.31)
	**Others**		
	65+	3	0.58 (0.12–1.69)
	65–74	1	0.68 (0.02–3.81)
	75–84	0	
	85+	2	1.85 (0.22–6.68)
**Stage**	**Localized**		
	65+	377	1.43 (1.29–1.58)
	65–74	111	1.52 (1.25–1.83)
	75–84	161	1.23 (1.04–1.43)
	85+	105	1.75 (1.43–2.11)
	**Regional**		
	65+	72	1.79 (1.40–2.25)
	65–74	12	1.25 (0.65–2.18)
	75–84	35	1.77 (1.23–2.46)
	85+	25	2.29 (1.48–3.39)
	**Distant**		
	65+	2	1.32 (0.16–4.79)
	65–74	0	
	75–84	0	
	85+	2	4.81 (0.58–17.38)
**Grade**	**Low**		
	65+	280	1.51 (1.34–1.70)
	65–74	88	1.61 (1.29–1.99)
	75–84	121	1.33 (1.10–1.59)
	85+	71	1.80 (1.40–2.26)
	**High**		
	65+	139	1.43 (1.21–1.69)
	65–74	28	1.28 (0.85–1.86)
	75–84	56	1.15 (0.87–1.50)
	85+	55	2.07 (1.56–2.69)
**Surgery**	**Yes**		
	65+	448	1.46 (1.33–1.61)
	65–74	123	1.48 (1.23–1.77)
	75–84	194	1.27 (1.10–1.47)
	85+	131	1.85 (1.55–2.19)
	**No Surgery**		
	65+	13	1.57 (0.84–2.68)
	65–74	4	1.78 (0.49–4.56)
	75–84	5	1.35 (0.44–3.14)
	85+	4	1.72 (0.47–4.42)

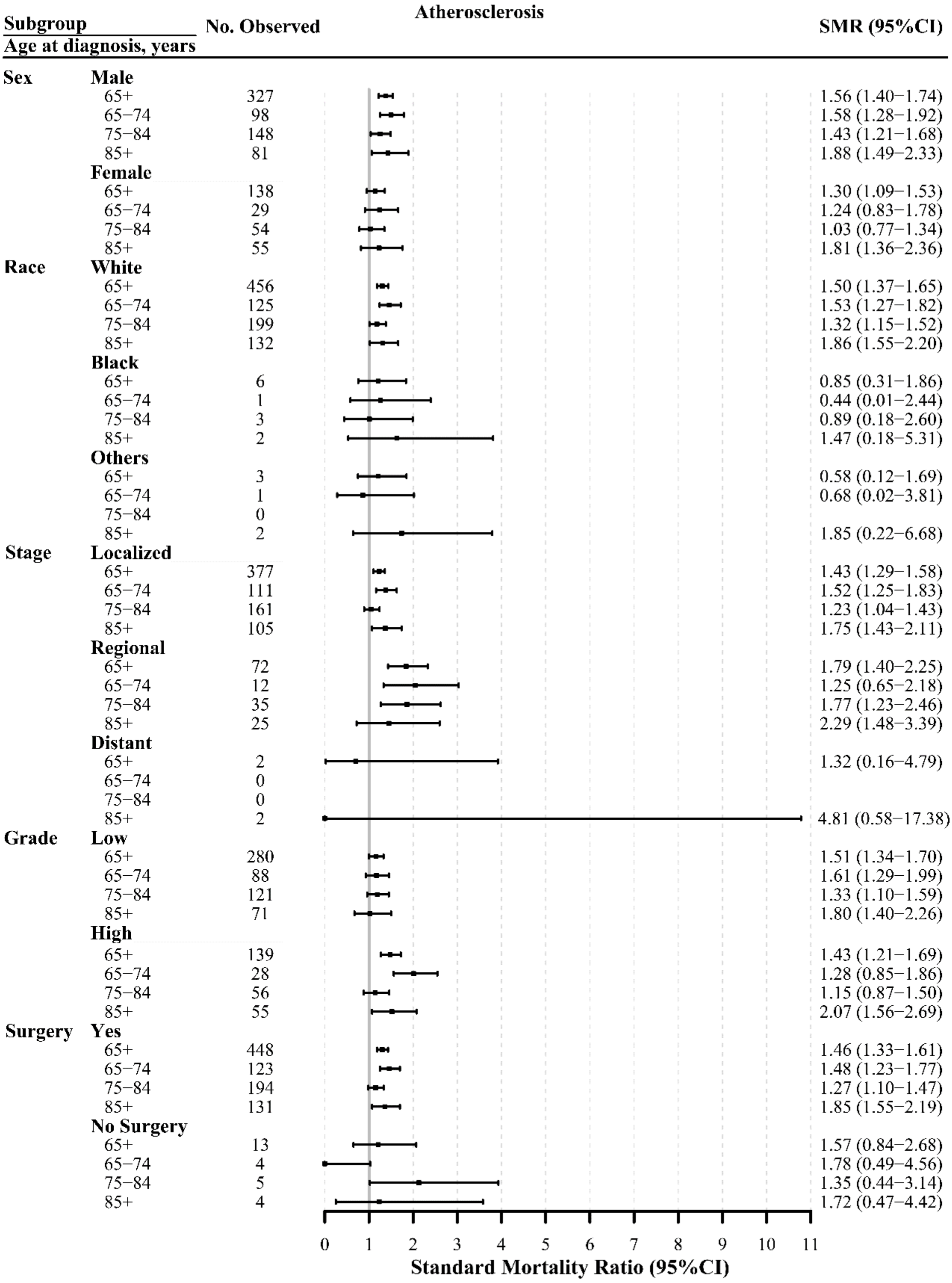

Supplementary Figure S11. The death risk of atherosclerosis in older patients (≥65 years) with bladder cancer based on age at diagnosis

Aortic Aneurysm and Dissection

Subgroup	Age at diagnosis, years	No. Observed	SMR (95%CI)
**Sex**	**Male**		
	65+	357	1.68 (1.51−1.87)
	65−74	177	1.79 (1.54−2.08)
	75−84	131	1.45 (1.21−1.72)
	85+	49	2.12 (1.57−2.81)
	**Female**		
	65+	66	1.49 (1.15−1.90)
	65−74	25	1.49 (0.96−2.20)
	75−84	30	1.50 (1.01−2.15)
	85+	11	1.47 (0.73−2.63)
**Race**	**White**		
	65+	400	1.63 (1.48−1.80)
	65−74	197	1.78 (1.54−2.05)
	75−84	152	1.44 (1.22−1.69)
	85+	51	1.75 (1.31−2.31)
	**Black**		
	65+	11	2.66 (1.33−4.77)
	65−74	5	2.58 (0.84−6.02)
	75−84	5	2.95 (0.96−6.88)
	85+	1	2.03 (0.05−11.29)
	**Others**		
	65+	12	1.66 (0.86−2.89)
	65−74	0	
	75−84	4	1.20 (0.33−3.08)
	85+	8	8.36 (3.61−16.48)
**Stage**	**Localized**		
	65+	357	1.64 (1.47−1.81)
	65−74	174	1.76 (1.51−2.04)
	75−84	134	1.43 (1.20−1.69)
	85+	49	1.90 (1.41−2.51)
	**Regional**		
	65+	60	1.92 (1.46−2.47)
	65−74	25	1.89 (1.22−2.79)
	75−84	25	1.81 (1.17−2.67)
	85+	10	2.35 (1.13−4.32)
	**Distant**		
	65+	1	0.80 (0.02−4.43)
	65−74	0	
	75−84	1	1.56 (0.04−8.69)
	85+	0	
**Grade**	**Low**		
	65+	246	1.66 (1.45−1.88)
	65−74	118	1.66 (1.37−1.98)
	75−84	102	1.64 (1.34−2.00)
	85+	26	1.70 (1.11−2.49)
	**High**		
	65+	140	1.72 (1.45−2.03)
	65−74	66	2.04 (1.58−2.59)
	75−84	48	1.29 (0.95−1.71)
	85+	26	2.19 (1.43−3.21)
**Surgery**	**Yes**		
	65+	406	1.63 (1.48−1.80)
	65−74	195	1.74 (1.51−2.00)
	75−84	157	1.47 (1.24−1.71)
	85+	54	1.84 (1.38−2.40)
	**No Surgery**		
	65+	15	2.10 (1.17−3.46)
	65−74	6	1.94 (0.71−4.23)
	75−84	3	1.01 (0.21−2.96)
	85+	6	5.42 (1.99−11.8)

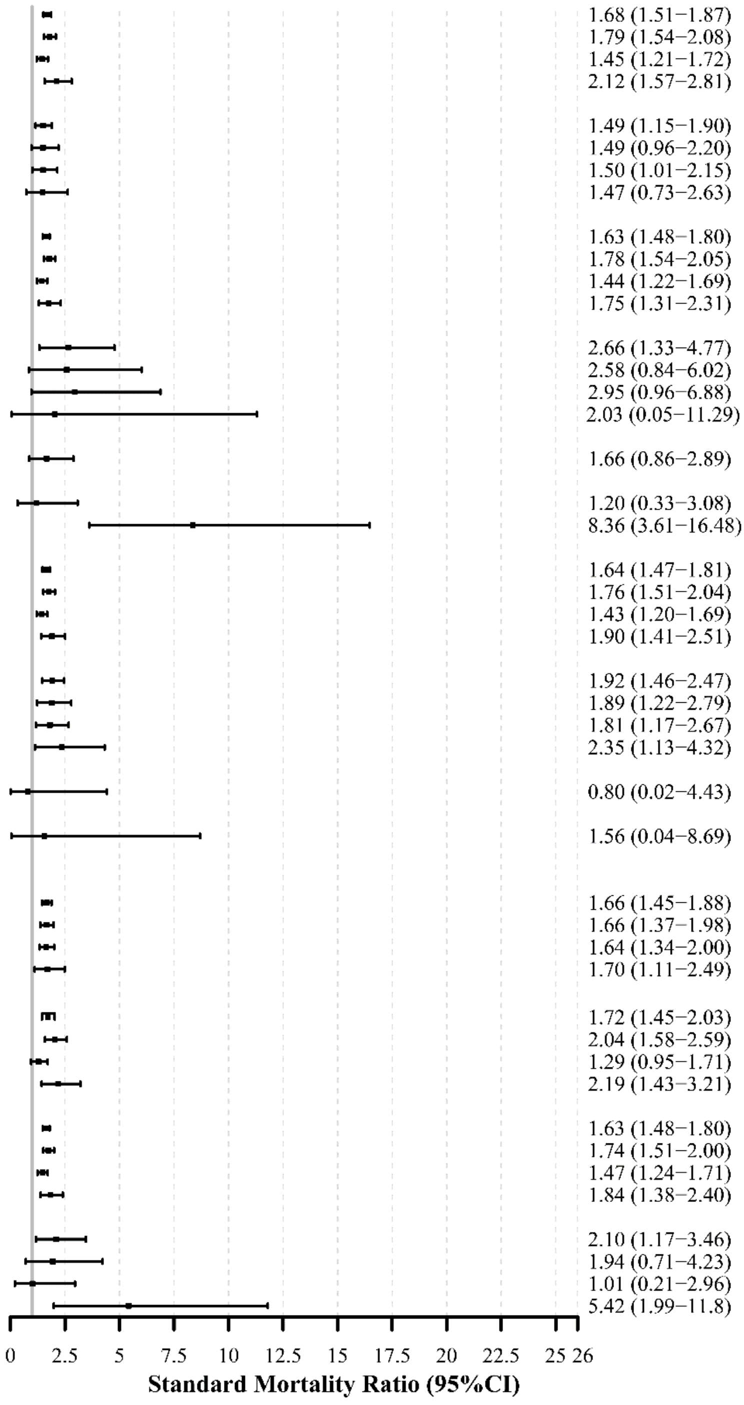

Supplementary Figure S12. The death risk of aortic aneurysm and dissection in older patients (≥65 years) with bladder cancer based on age at diagnosis

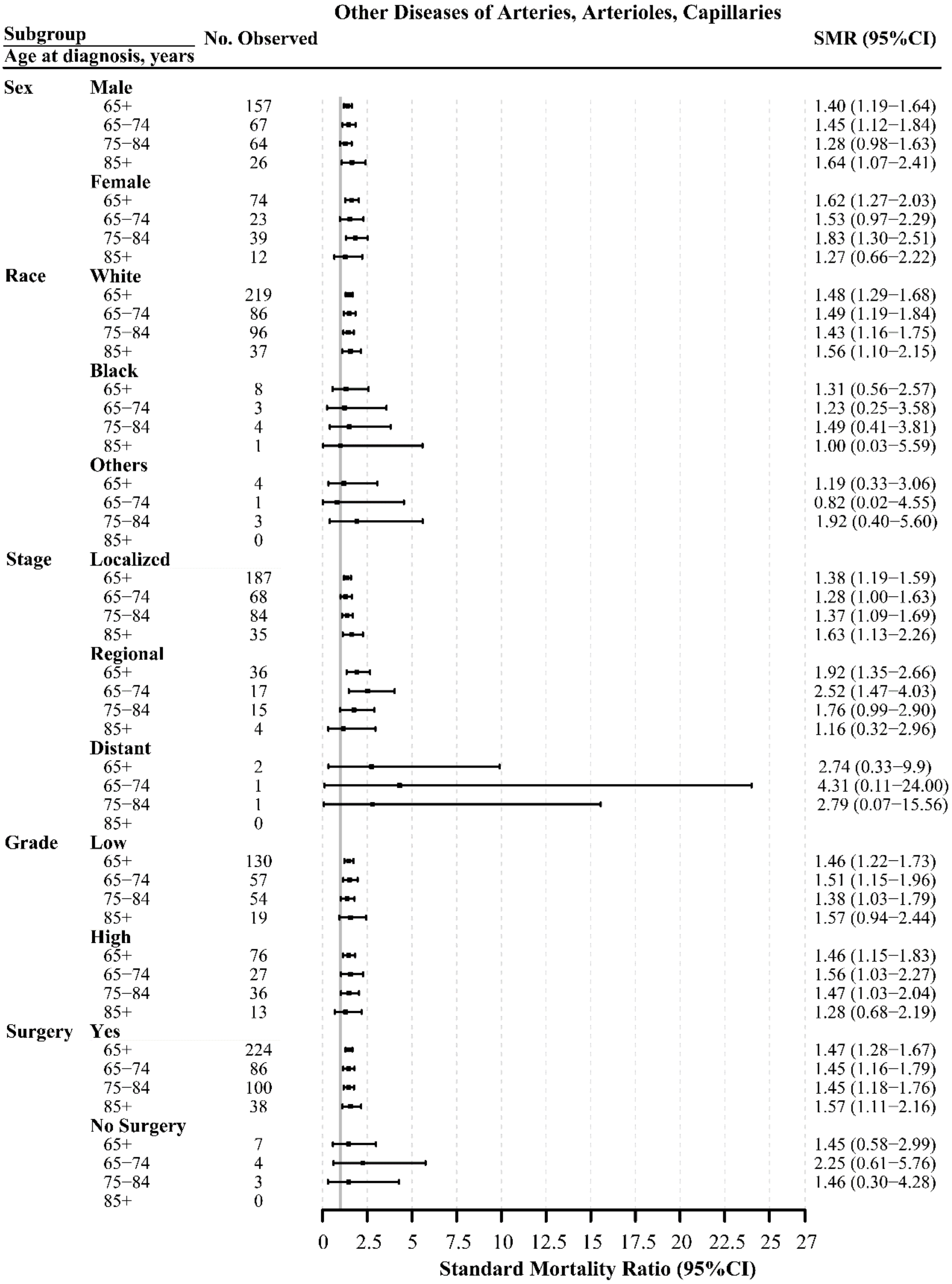

Supplementary Figure S13. The death risk of other diseases of arteries, arterioles, capillaries in older patients (≥65 years) with bladder cancer based on age at diagnosis
